# 170 Years of “Lock-and-Key”: Genital Morphology and Reproductive Isolation

**DOI:** 10.1155/2012/247352

**Published:** 2011-12-10

**Authors:** John P. Masly

**Affiliations:** Department of Zoology, University of Oklahoma, 730 Van Vleet Oval, Norman, OK 73019, USA

## Abstract

The divergent genital morphology observed among closely related animal species has long been posited as a mechanism of reproductive isolation. Despite the intuitive appeal that rapidly evolving genitalia might cause speciation, evidence for its importance—or even its potential—in reproductive isolation is mixed. Most tests of genital structural isolation between species often fail to find convincing evidence that differences in morphology prevent copulation or insemination between species. However, recent work suggests that differences in genital morphology might contribute to reproductive isolation in less obvious ways through interactions with sensory mechanisms that result in lowered reproductive fitness in heterospecific matings. In this paper, I present a brief history of the “lock-and-key” hypothesis, summarize the evidence for the involvement of genital morphology in different mechanisms of reproductive isolation, discuss progress in identifying the molecular and genetic bases of species differences in genital morphology, and discuss prospects for future work on the role of genitalia in speciation.

*L'armure copulatrice est un organe ou mieux un instrument ingénieusement compliqué, destiné à s'adapter aux parties sexuelles externes de la femelle pour l'accomplissement de l'acte copulatif; elle est la garantie de la conservation des types, la sauvegarde de la légitimité de l'espèce. [The copulation armor is an organ or better an instrument ingeniously complicated, destined to adapt to sexual parts external to the female for the completion of copulation; it is the guarantee of the preservation of the standards, the safeguard of the legitimacy of the species.]*

L. Dufour, 1844

## 1. Introduction

The French entomologist Leon Dufour's statement [1] in which he hypothesized that the remarkable diversity in genital morphology observed among Dipterans is important for maintaining reproductive isolation (RI) between species is one of the notable ideas in speciation research that has generated both considerable controversy and much experimental work. Indeed, among different mechanisms of RI, structural isolation involving genitalia appears to be one of the first mechanisms of speciation to be experimentally tested. However, despite the widespread differences in genital morphology observed among many animal species [2], and the intuitive nature of the so-called lock-and-key hypothesis—that structural differences in the genitalia prevent species from hybridizing—the vast majority of experimental tests have failed to find convincing evidence that the differences in genitalia between species have a substantial role in preventing hybridization. Most of these tests, however, have focused on one specific mechanism of genital lock-and-key RI, and recent studies suggest that differences in genital morphology might in fact contribute to RI in more cryptic ways that reduce the reproductive success of heterospecific matings. 

Lock-and-key reproductive isolation can operate via two different mechanisms [3, 4]. The first is the classic mechanical or structural lock-and-key mechanism of Dufour where differences in genital morphology between species prevent or reduce the success of copulation and/or insemination as a direct result of mechanical incompatibilities that occur during genital coupling. RI caused by structural lock-and-key also includes postmating fitness losses such as physical damage to either parent that might prevent any future matings. The second mechanism is sensory lock-and-key first posited by De Wilde [5] and later formalized by Eberhard [3] where differences in genital morphology are perceived by one or both sexes and evoke behavioral or physiological responses that result in premature termination of mating attempts or postcopulatory reproductive fitness problems. These two mechanisms are not mutually exclusive, and both can operate in concert to give rise to RI. 

Because genital morphology and its role in RI have received much attention in literature, it will be helpful to make clear what I will discuss in this review. Several hypotheses about the forces that drive the rapid evolution of genital morphology in animal taxa have been put forth, and there is good evidence that sexual selection often operates to drive the evolution of genital morphology within species. These hypotheses and the data supporting them have been reviewed thoroughly elsewhere [2, 6–8], and I refrain from doing so here. I also refrain from reviewing the evidence testing genital lock-and-key RI using comparative or phylogeographic data. My reason for doing this is that without detailed knowledge of the evolutionary histories of species, it is often difficult to draw firm conclusions about the role of genitalia in RI from these patterns alone. For example, although patterns like reproductive character displacement are consistent with reinforcement acting on genital morphology where species exist either sympatrically or parapatrically, an absence of reproductive character displacement does not preclude a history of differences in genital morphology contributing to RI between species, as other RI mechanisms (e.g., mate discrimination, ecological niche divergence, temporal isolation, etc.) might evolve via reinforcement before reproductive character displacement might evolve. 

Instead, my goal here is to examine evidence of hybridizations between species where differences in genital morphology appear to contribute to RI either by structural lock-and-key and/or sensory lock-and-key mechanisms. Although many studies exist that present indirect evidence to suggest a possible role for genitalia in contributing to RI between species, I focus my discussion on only those studies that have either directly observed species crosses in nature, or performed laboratory crosses between species, and recorded postcopulatory reproductive fitness in heterospecific pairs. In each of these cases it is important to emphasize that other mechanisms of RI beside genital lock-and-key also exist between these species pairs, and I do not suggest that genital isolation is the primary cause of speciation in any of these examples. Rather, I present these data to illustrate the possibility that RI between species can occur as a byproduct of differences in genital morphologies that have evolved in response to evolutionary forces such as sexual selection acting within species. In describing each of the heterospecific crosses below, I use the convention of always presenting the female parental species first (e.g., *species* A female × *species* B male).

## 2. The History of the Idea

Observations of genitalia and structural isolation have enjoyed a long history in the study of speciation and systematics (for a detailed history of the structural lock-and-key hypothesis and tests of the hypothesis, see [9]). Much of the appeal of the structural lock-and-key hypothesis is that it offers an intuitively obvious mechanism of RI and an appealing visual image. The idea that incompatibilities between genitalia of different species caused RI was perhaps considered so obvious that it appears few careful tests of structural isolation were performed in the years following Dufour. Among some of the early proponents of the idea was one of Dufour's students, Pérez [10], who made similar observations that genital morphology among several Hymenoptera species was incredibly diverse, and supported the notion that these differences in male genital morphology were the likely cause of RI among species (Although Pérez seemed to accept that differences genital morphology are important for RI, he does urge some caution in his interpretation as he notes that in some groups the genital morphology among males varies very little. It is a bit unclear, however, if his caution is in regard to the potential for genitalia to cause RI, or for their usefulness in systematic classification.). Another early champion of the idea, and one of the first to make careful observations of genital coupling between males and females in a laboratory setting, was Jordan [11]. Jordan performed crosses between species of *Papilio* and observed that for crosses within species, the male and female external genitalia are well-matched and fit together with tight coupling, but in crosses between species, the coupling between the male and female genitalia is not quite as good. Specifically, Jordan found that although the external genitalia of *Papilio memnon* males and females fit to provide tight genital coupling, in crosses between *P*. *helenus* and *P*. *memnon* and between *P*. *podalirius* (the modern classification of this species is *Iphiclides podalirius*) and *P*. *machaon*, the male and female genitalia did not fit as precisely to secure the copulating pairs as they did within species. Jordan also observed that, similar to males, the females of different species also possess genital structures that appear different, but these differences are not as dramatic as those observed among male genital structures. 

Despite these early observations that seemed to support structural lock-and-key RI, many subsequent observations called the ubiquity, and even the existence, of RI as a result of differences in genital morphology into question. Boulangé [12] pointed out that in many Hymenoptera species, the most divergent structures of the male genitalia are those that are *not* involved in intromission and insemination. Instead, these structures make contact with female structures that possess essentially identical morphology across species, for example, the sides of the female abdomen. He also observed that in comparisons between species where females do possess divergent genital morphology, the differences are usually minor. Richards [13] and later Robson and Richards [14] made several observations in *Bombus* (subgenus *P sithyrus*) that also did not support the structural lock-and-key hypothesis. Among the many criticisms they level against structural lock-and-key, they found that females usually do not differ greatly in genital morphology, so the required structural “locks” simply do not exist, the morphological differences in male genitalia of some species are so small as to likely have no effect on reducing the tight coupling between male and female genitalia, and there appears to be no correlation between the degree of diversity in genital morphology among species and the species richness of most groups. Robson and Richards also presented what is perhaps the most serious criticism against the importance of structural lock-and-key in RI: species that possess dramatic differences in genital morphology can often mate and produce offspring. From these observations, they state with regard to RI, “we are forced to regard specific differences in the genitalia as of essentially the same nature as other apparently useless specific characters” [14, page 297]. They conclude that divergent genital morphologies are more likely the result of RI rather than its cause, and other isolating mechanisms (in particular mate discrimination) probably play more important roles in speciation. For many years following, tests for genital structural isolation were performed in a variety of animal taxa and almost all of these tests failed to find convincing evidence of structural lock-and-key RI [9]. 

What exactly “lock-and-key RI” implies has also become a bit muddled throughout the literature since the introduction of the idea. A common notion of lock-and-key RI describes the phenomenon as natural selection acting on genital morphology *to prevent species from hybridizing*. When considering the case of reinforcement, this is certainly an applicable definition, but as a more general definition to describe genital lock-and-key RI, it is incorrect. Just as some other RI mechanisms such as intrinsic postzygotic isolation evolve as a byproduct of evolutionary processes that occur within populations evolving in isolation, genital lock-and-key RI can also evolve as a byproduct of evolutionary processes that occur within isolated populations, such as sexual selection, that can act to drive genital morphological evolution. The divergence in morphology between two populations might consequently give rise to either structural or sensory isolation as a byproduct when they attempt to hybridize. A history of sympatry or parapatry is not required for lock-and-key RI to evolve under this scenario, and I use this broader definition of lock-and-key in evaluating the contribution of genitalia to RI.

## 3. Structural Isolation

For structural isolation to operate two criteria need to be satisfied. First, the genitalia of both males and females are required to bear substantial, species-specific differences in morphology of structures important for successful copulation, intromission, and/or insemination. Second, male and female genitalia *within* species are required to show strong correlated evolution in the morphology of these structures. Tests of structural isolation are often difficult as they require both incomplete premating RI between species and careful measurements of parental fitness loss after heterospecific matings. Despite these challenges, crosses between species in a handful of arthropod and arachnid taxa suggests that structural lock-and-key may, in fact, contribute to RI in at least some hybridizations. 

Standfuss [15, pages 60-61] observed crosses between 24 heterospecific pairs of the lackey moths *Bombyx franconica* and *B. neustria* (modern day classifications for these two species are *Malacosoma franconica* and *M. neustria*, resp.). This large number of heterospecific matings suggests that little premating isolation exists between these two species, and indeed, courtship and copulation appear to proceed normally [15]. After mating, the *M. franconica* females also appear to exhibit the normal postcopulatory behavior by walking around to identify locations to oviposit. Within the next three to four hours of observation, however, most of these females die. Among those that survive, some are unable to lay eggs. Others do lay eggs, but egg lethality ranges from 50–100% and larvae never reach adulthood. Although no description is given of the morphological differences in male or female genitalia between these two species, Standfuss speculates that the probable cause of lethality of *M. franconica* females was injuries suffered as a result of the *M. neustria* male genitalia. He is, however, cautious in his interpretation, and hypothesizes that other species differences might have been the cause of female lethality (In a remarkably insightful passage for the time period, Standfuss hypothesizes that some of the other possible causes of egg mortality and female lethality might include sperm-egg incompatibility and what he describes as “molecular differences” between the two species.).

Structural isolation also appears to operate in some other species of Lepidoptera. Federley [16, pages 371-372] performed crosses between the hawk moths *Metropsilus porcellus* and *Chaerocampa elpenor* (modern day classifications for these two species are *Deilephia porcellus *and *D. elpenor*, resp.). Upon intromission, *D*. *elpenor* males become “stuck” and are unable to withdraw their aedeagus from the *D*. *porcellus* females. In the case of copulating species pairs that do manage to separate, females never lay eggs, although sperm is found in the bursa copulatrix, which suggests the possibility that damage to the female reproductive tract might prevent fertilization and/or oviposition. Males of these two species bear substantial morphological differences in their external genitalia: *D*. *elpenor* males possess a long and slender aedeagus, whereas *D*. *porcellus* males possess a shorter and thicker aedeagus compared to those of *D*. *elpenor* males. These differences in male genitalia are consistent with structural isolation in the *D*. *porcellus* × *D*. *elpenor* cross, and also agree with the results from the reciprocal cross—*D*. *elpenor* × *D*. *porcellus* proceeds normally through intromission and insemination [16]. 

Males and females of several species of crab spider in the genus *Misumenops *possess genital morphologies that are both highly divergent among species and show strong correlated evolution between males and females within species [17]. During copulation, the male intromittant organ, the palpus, is guided into the reproductive tract by the female epigynum to successfully complete intromission and insemination. In the cross *M. rothi* × *M. gabrielensis*, *M. gabrielensis* males initiate courtship and are received by the female, but are unable to orient their palpus and complete intromission because the *M. gabrielensis* palpus is much larger than the opening to the epigynum in *M. rothi*. (It seems possible that the reciprocal cross might proceed through intromission and insemination, but was not attempted [17].) In the cross *M. lepidus* × *M. californicus* attempts at intromission were unsuccessful because the *M. californicus* male failed to correctly align the palpus with the epigynal structures of the *M. lepidus* female presumably because of the structural incompatibilities between the two structures. The reciprocal cross results in successful intromission and insemination, but does not produce offspring. Individual females have been collected from nature that show genital morphology intermediate to those of *M. lepidus* and *M. californicus*, thus it does appear that the structural RI in the *M. lepidus* × *M. californicus* cross is incomplete [17]. 

**Figure 1:**
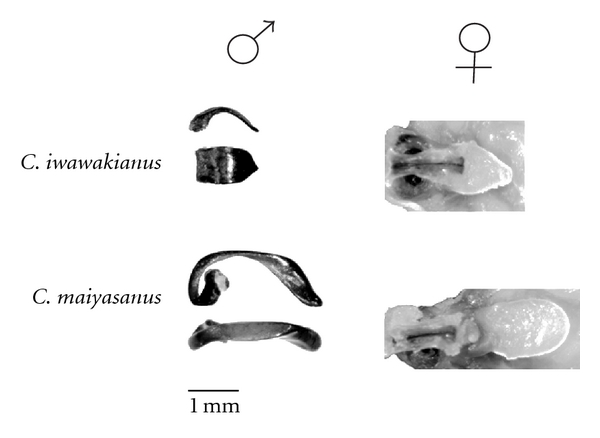
Genital morphology in *Carabus iwawakianus* and *C. maiyasanus*. The male copulatory piece is shown from both dorsal and sagittal views. The female vaginal appendix for each species is also shown. Images courtesy of Teiji Sota.

One of the best-characterized examples of structural lock-and-key comes from hybridizations among beetles of the genus *Carabus* (subgenus *Ohomopterus*) [18]. *Carabus *comprises a large group of wingless beetles on Japanese island of Honshu. Many species in the genus exist parapatrically and form hybrid zones. In the *C. iwawakianus* and *C. maiyasanus* species pair, males of both species do not discriminate against heterospecific females, and hybrid individuals that possess intermediate genital morphology between these two species have been found at low frequencies in the hybrid zone. Males of each species possess striking differences in genital morphology: *C. iwawakianus* possesses a short and wide copulatory piece, whereas *C. maiysanus* possesses a long and thin copulatory piece (Figure 1). The vaginal appendix of the females in each species also shows striking correlated morphology with the copulatory piece of their conspecific males (Figure 1). During intromission, the copulatory piece of the male and vaginal appendix female lock together. Sota and Kubota [18] performed reciprocal crosses between these two species and measured male and female fitness after intromission. In the cross *C. iwawakianus* × *C. maiyasanus *50% (18 of 36) of *C. maiyasanus* males suffer broken copulatory pieces that were likely to prevent future matings. Female *C. iwawakianus* mortality is high (60%, 20 days postmating) and postmortem dissections revealed ruptured vaginal appendices and torn bursae in the majority of cases. In the reciprocal cross *C. maiyasanus* × *C. iwawakianus*, none of the *C. iwawakianus* males suffer injuries to the copulatory piece (0 of 27), and female *C. maiyasanus* mortality is lower compared to the *C. iwawakianus* × *C. maiyasanus* cross (~30% versus 60%). However, a substantial fraction of *C. maiyasanus* females still suffer damage to the vaginal appendix that appear severe enough to prevent future mating. In both directions of crosses between these species, mated females lay fewer eggs and the egg hatch rates are lower, although it is unclear whether this is a direct consequence of damage to the female reproductive tract by the male genitalia. Structural isolation might prove to be a common mechanism of RI among *Carabus* species as female mortality following heterospecific crosses has also been reported between *C. albrechti* and *C. iwawakianus* [19] and between *C. albrechti* and *C. yamato* [20]. 

 Another well-characterized example of a species group where structural lock-and-key appears to have an important role in RI occurs among some species of millipedes [21]. The *Parafontaria tonominea* species complex are also endemic to Japan, and many species exist parapatrically (some species exist sympatrically). Tanabe and Sota [21] performed crosses between *Parafontaria tonominea* sp. A and *Parafontaria tonominea* sp. B., two species that differ in their overall genital morphology, particularly in the size of their genital structures. They also possess notable differences in body size with *Parafontaria* sp. A possessing a larger body size than *Parafontaria* sp. B. Courtship and intromission between these species requires multiple steps and is highly choreographed. Courtship is initiated by the male with antennal contact, the male aligns head-to-head with female, and a preliminary intromission occurs without sperm transfer. If the female remains receptive following preliminary intromission, true intromission and insemination occur, which is then followed by postcopulatory behavior by the male. When preliminary intromission fails the male aborts the mating attempt. No apparent premating isolation exists between *Parafontaria* sp. A and *Parafontaria* sp. B, as males are equally likely to mate with a female of either species [21]. 

In the cross *Parafontaria* sp. B × *Parafontaria* sp. A, preliminary intromission fails in every cross (10 of 10) despite repeated contact between the male and female genitalia. The cause of the failed intromission is the difference in size between the much larger *Parafontaria* sp. A male gonopod and the smaller *Parafontaria* sp. B female gonopore. In the reciprocal cross *Parafontaria *sp. A × *Parafontaria* sp. B, preliminary intromission fails in ~65% of the attempts (9 of 14) because the smaller *Parafontaria* sp. B male is unable to align his gonopod correctly even after repeated attempts due to his smaller overall body size. Four of the five remaining *Parafontaria* sp. A × *Parafontaria* sp. B crosses resulted in successful preliminary intromission, and true intromission occurred in three of the five. In each of these cases, however, the *Parafontaria* sp. B male terminated postcopulatory behavior prematurely. 

A recent study has also identified structural isolation between two members of the *Drosophila melanogaster* species subgroup. *D. yakuba* and *D. santomea* inhabit the island of Saõ Tomé off the western coast of Africa. Although these two species occur primarily at different elevations, their ranges overlap and form a hybrid zone at mid-elevation locations around the island [22, 23]. *D. yakuba* males possess a pair of sclerotized spikes that project from the lateral portion of the aedeagus. *D. santomea* males also possess sclerotized projections in the same location, but the morphology of the projections is rounded and more “nub-like.” Females of each of these species show correlated evolution of genital structures with those of their conspecific males: *D. yakuba* females possess a pair of heavily sclerotized cavities that receive the male aedeagus spikes during copulation, whereas *D. santomea* females lack these cavities. 

Kamimura and Mitsumoto [24] studied the consequences of these morphological differences on structural isolation between *D. santomea* and *D. yakuba*. In the cross *D. santomea* × *D. yakuba*, *D. yakuba* males successfully mount the *D. santomea* females, but usually fail to insert their aedeagus during mating, which results in insemination of only 20% (3 of 15) of the females in heterospecific matings. In 11 of the 12 remaining crosses that did not result in insemination, the male ejaculate was observed as a white, sperm-bearing mass on the external genitalia of either the *D. yakuba* male or *D. santomea* female in each pair. This mass appears to bind the genitalia of mating pairs together as heterospecific pairs are often observed struggling to separate from each other. Moreover, severe copulatory injuries were observed in mated *D. santomea* females that match the pattern of aedeagus spikes from the *D. yakuba* male. The reciprocal cross *D. yakuba* × *D. santomea* proceeds through copulation and insemination, although it is easy to dislodge mating pairs, which suggests that the spikes in *D. yakuba* may have evolved to secure genital coupling [24]. Similar modifications of male genitalia that function to secure genitalia during copulation within species have also been documented in *D. bipectinata* [25].

## 4. Sensory Isolation

*It has been thought that the highly specific shape of structures and appendages of male and female copulatory apparatus constitutes a decisive structural factor in species isolation, acting as a system of key and lock. But it would rather seem that intraspecific matings are assured by precopulatory behavior, and probably by the mutual stimulation of specific sensory sites during the copulatory act.*

J. De Wilde, 1964

The possibility that genital incompatibilities might cause RI between species through behavioral or physiological responses has recently received renewed interest. In contrast to the requirements for structural lock-and-key RI where both male and female genital structures are required to possess both complementary morphologies within species and divergent morphologies between species, sensory lock-and-key RI requires that only *one *of the sexes possesses divergent morphology between species. The morphology of the opposite sex, whether species-specific or not, requires innervation with sensory neurons capable of relaying information about species identity. Compared to structural lock-and-key mechanisms, sensory lock-and-key is perhaps more difficult to detect for the primary reason that the phenotype being studied is more complex—rather than studying a simple structural incompatibility, it is necessary to study a structurally induced behavior, or structurally induced changes in physiology. Although RI via sensory lock-and-key has not been as extensively studied as structural lock-and-key, there is growing evidence that it operates in some species crosses. 

In laboratory crosses among several genera of Lepidoptera, forced matings often show no evidence of structural lock-and-key preventing successful insemination in heterospecific matings, despite that fact that male genital morphology often differs dramatically among closely related species (e.g., [26]). Although differences in male genital morphology do not appear to hinder the mechanics of copulation and intromission, they may relay species identity between the sexes. Two species of brush-footed butterflies *Erebia nivalis* and *E. cassioides* form hybrid zones along altitudinal gradients at some locations in the Alps. Although strong mating discrimination exists between them, heterospecific crosses can be obtained in the laboratory [26]. In conspecific crosses, mating pairs will remain in copula ~18–30 minutes after a male successfully courts a female. However, in the heterospecific cross *E. nivalis* × *E. cassioides*, the male terminates copulation after only 0.5–7 minutes [27]. During this time the female remains receptive to the heterospecific male, and presumably the male perceives the female is heterospecific and terminates copulation prematurely, although it is also possible that the female fails to cooperate with the male and as a result he terminates the mating attempt. The duration of copulation in the reciprocal cross *E. cassioides* × *E. nivalis* appears normal [27]. 

Another example sensory lock-and-key occurs among some scarab beetle species [3]. *Macrodactylus costulatus*, *M. sylphis*, and *M. sericinus* females each possess relatively similar genital morphologies, but males of these species possess substantial differences in their genitalia. The differences in male morphology are most pronounced not in the hard cuticular structures of the external genitalia, but rather in the soft tissues involved in intromission. Among these three species, Eberhard [3] observed 37 male mounting attempts between heterospecific pairs out of total 160 mounting attempts in the field. All attempts to mount by heterospecific males were short (<5 seconds) and intromission never occurred. *Macrodactylus* females are known to reject unwanted males in conspecific crosses by contracting their vaginal walls to prevent intromission [28], thus it seems likely that females perceive heterospecific males and reject their copulation attempts using a similar behavior. However, it also seems possible that because the male morphological differences occur in soft and possibly sensory tissues, the male might perceive species identity of females via these differences in genital morphology, and terminate mating attempts prematurely.

Damselflies present an interesting case where both structural and sensory lock-and-key mechanisms appear to contribute to RI. During courtship, the male damselfly grasps the female with his legs and brings his terminal appendages into contact with two mesostigmal grooves on the back of the female thorax. Both the male appendages and the female mesostigmal grooves within species show structural complementary and species-specific morphologies [29, 30]. Ablation experiments show that the male terminal appendages are important for mate recognition and copulation; females perceive the superior appendages, whereas the inferior appendages are used primarily by males for grasping the female [30]. Unreceptive females resist copulation by vigorously beating their wings and orienting their abdomen to prevent the males from achieving genital coupling. If the female is receptive, she bends her abdomen to make contact between her genitalia and those of the male. When damselflies occur in large groups, males will usually mate with conspecific females, but heterospecific matings do occur in nature [31]. 

In several genera of damselflies, the fit between the male appendages and the female mesostigmal grooves appears to be used by the female to assess species identity of the male. In heterospecific crosses among different *Sympecma* and *Lestes* species, heterospecific females vigorously resist copulation shortly after the male grasps the female and makes contact with his terminal appendages [31]. Similar observations have also been made in crosses between *Ischnura elegans* males mated to females of species belonging to different damselfly genera [32]. Paulson [33] performed several laboratory crosses among *Enallagma, Argia, Ischnura*, and *Telebasis* species. He observed that in contrast to conspecific pairs, in heterospecific pairs, the male appendages are unable to grasp the female thorax correctly. Roughly 66% of the crosses (8 of 12) show nearly complete inability of grasping by heterospecific males, which suggests structural incompatibilities between the morphologies of the male appendages and the female mesostigmal grooves also contribute along with sensory isolation to RI among these species. 

**Figure 2:**
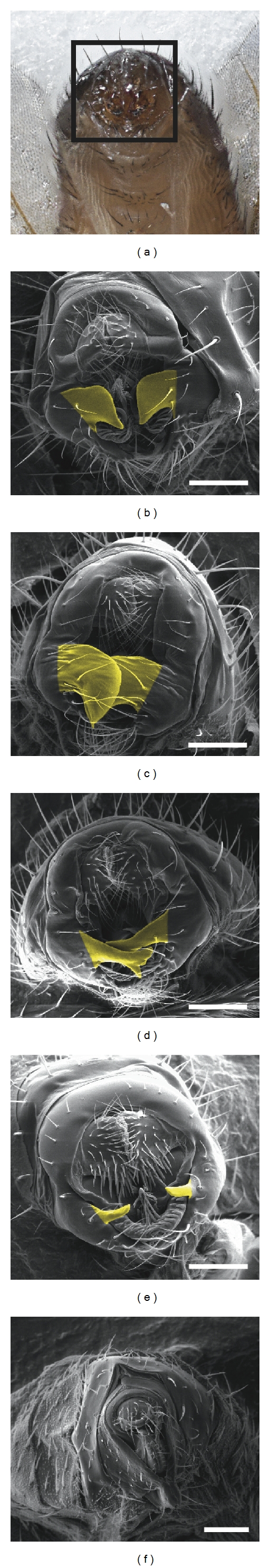
External genital morphology among members of the *Drosophila melanogaster* species complex. (a) Terminal portion of the male abdomen representative of the four members of the *D. melanogaster *species complex. Black box denotes the area of male genitalia shown in the scanning electron micrographs presented in panels (b–e). (b) *D. melanogaster* male, (c) *D. simulans* male, (d) *D. sechellia* male, (e) *D. mauritiana* male. Yellow shading marks the posterior lobe of the genital arch. (f) Female genital morphology representative of members of the *D. melanogaster *species complex. Scale bars are 100 *μ*m.

Evidence of sensory lock-and-key is also found in some *Drosophila* species. Among members of the *D. melanogaster* species complex (*D. mauritiana, D. melanogaster, D. sechellia, D. simulans*), males possess two bilaterally symmetrical sclerotized cuticular genital structures called the posterior lobes of the genital arch, which insert between the eight and ninth tergites of the female during copulation [34], and differ dramatically in both size and shape among species (Figure 2; [35, 36]). Females of these species show no apparent differences in external genital morphology, although the posterior lobe likely comes into contact with, or distends soft abdominal tissues during copulation. No evidence exists of a structural lock-and-key mechanism among the *D. melanogaster* complex species that involves the posterior lobe, but the posterior lobe does appear important for mounting and genital coupling [37, 38]. (It is worth pointing out, however, that the posterior lobe in *D. sechellia* and *D. simulans* is known to cause some damage to the soft abdominal tissues at the insertion site in conspecific crosses [39].)

 Heterospecific crosses among *D. mauritiana, D. sechellia,* and *D. simulans* show two cryptic defects that reduce the reproductive success of heterospecific pairs. The first defect is that copulation is generally shorter when females mate with heterospecific males compared to copulation duration when females mate with conspecific males. Within species, copulation duration lasts an average of *∼*15–17 minutes in *D. mauritiana*, *∼*30 minutes in *D. sechellia*, and *∼*25–30 minutes in *D. simulans* [37, 40, 41]. In the *D. simulans* × *D. mauritiana* cross, copulation is much shorter than either pure species cross and lasts only 5–11 minutes [37, 40–42]. During copulation, the *D. simulans* females also actively resist mounting attempts of *D. mauritiana* males. In the *D. mauritiana* × *D. simulans* cross, copulation duration is slightly shorter than that of *D. simulans* heterospecific crosses, but abnormally long compared to *D. mauritiana* conspecific crosses [40]. In the *D. simulans* × *D. sechellia* cross, copulation duration varies from as short as ~16 minutes to the normal *∼*25–30 minutes observed in *D. sechellia* conspecific crosses. In each heterospecific pair (including heterospecific crosses involving the other species in the group, *D. melanogaster*; [38]), the duration of copulation is more similar to the copulation duration typical of the male species. This suggests the possibility that differences in posterior lobe morphology might allow males to maintain copulation duration for times that are characteristic of that species, which could be important for successful insemination [38]. 

The second defect observed among these heterospecific crosses is abnormal sperm transfer and lower offspring production. In the *D. simulans *× *D. mauritiana* cross, a smaller fraction of the *D. mauritiana* sperm are stored by the *D. simulans* female compared to either pure species cross, and the cross produces 40% fewer offspring compared to pure species *D. mauritiana* and 70% fewer compared to pure species *D. simulans* [37, 40]. In the *D. mauritiana* × *D. simulans* cross, *D. simulans* males transfer an abnormally large number of sperm during copulation, however, the female loses the heterospecific sperm rapidly from her storage organs. Oviposition rates of mated females are reduced, and the cross yields fewer progeny [40]. Lastly, in the *D. simulans* × *D. sechellia* cross, *D. sechellia* males transfer few or no sperm to *D. simulans* females even when copulation duration last the full 30 minutes [40]. Although the morphology of the posterior lobe might contribute to these postinsemination reproductive defects, these reproductive fitness problems might also result from differences in molecular incompatibilities between male seminal fluid proteins and proteins in the female reproductive tract, which are known to cause postcopulatory reproductive problems between other *Drosophila* species [43, 44].

## 5. Genetics of Species Genital Morphology

Because of their rapidly evolving morphology and their importance in reproductive fitness, animal genitalia have attracted the attention of evolutionary geneticists. Aside from presenting a good model to study the genetics of rapidly evolving morphological traits, there is reason to suspect that genitalia might possess a particular genetic architecture if sexual selection drives the evolution of morphology within species [4]. In particular, we might expect that several genes would be necessary to specify differences in genital morphology (or in the case of sensory lock-and-key, differences in behavioral preferences) in both males and females, reflective of the step-wise coevolution of phenotypes between the sexes. We might also predict to find the molecular signature of selection at loci important for specifying morphological (behavioral) differences. 

Many closely related species possess divergent genital structures thus making it possible to construct interspecific hybrid genotypes to dissect the genetics of genital morphology. Although experiments mapping differences in genital morphology have been performed in only two species groups where genitalia contribute to RI, the results of these mapping experiments suggest that the genetic architecture of species differences in genital morphology bears some similar characteristics across taxa. The results also support at least one of our predictions: species differences in genital morphology appear to be specified by many genes, and the phenotypic effects of species alleles act in the direction of the species phenotype, consistent with the idea that sexual selection drives the evolution of morphology within species. Moreover, the genomic locations that have large effects on morphological differences are similar among species within some species groups, which suggests the possibility that change at some of the same loci might be involved in the evolution of species-specific genital morphology. 

Recent work on *C. maiyasanus* and *C. iwawakianus* has identified several genomic regions between these two species that carry loci specifying the species differences in both the male and female genital morphology involved in structural lock-and-key RI [45, 46]. Sasabe and colleagues measured a panel of reciprocal F_1_ and backcross hybrids for genital morphology and performed quantitative trait loci (QTL) mapping experiments to identify the minimum number of genes that specify male and female morphology. They measured two genital phenotypes for each sex: in males, they measured copulatory piece length and copulatory piece width, and in females they measured vaginal appendix length and vaginal appendix width. The results of their mapping identified 15 QTL that reside across 8 of the 14 linkage groups in these species: three QTL specify differences in copulatory piece length, three QTL for copulatory piece width, four QTL for vaginal appendix length, and five QTL for vaginal appendix width. 

Several genetic studies have also been performed that map the genomic regions that specify the differences in posterior lobe morphology among the *D. melanogaster* complex species. Because crosses to *D. melanogaster* usually result in dead or sterile offspring [47, 48], most of these mapping studies focus on comparisons among *D. mauritiana*, *D. sechellia*, and *D. simulans* where it is easier to obtain F_2_ and backcross hybrid genotypes [49]. QTL mapping experiments between *D. mauritiana* and *D. simulans* identified 20 QTL that map across each of the major chromosomes in these species underlying the posterior lobe morphological differences [50]. In the *D. sechellia*-*D. simulans* species pair, QTL mapping revealed a minimum of 13 QTL that have effects on posterior lobe morphology [51]. An introgression mapping approach was used to map loci specifying morphology between *D. mauritiana* and *D. sechellia* to small genomic regions across roughly 50% of the genome. The mapping results identified a minimum of six regions with large effects on morphology [52]. Interestingly, some of these genetic regions have morphological effects on posterior lobe size, but not posterior lobe shape, whereas others have morphological effects on posterior lobe shape, but not posterior lobe size. This result suggests that these two posterior lobe phenotypes are specified, in part, by different loci. Transcriptome sequencing experiments in the larval tissue that gives rise to the male genitalia also reveal a possible role for gene expression differences in the insulin/insulin-like signaling pathway in specifying morphological differences between *D. mauritiana* and *D. sechellia* [52].

## 6. Conclusions and Prospects

Although it has long been thought that differences in genital morphology had little or no importance for speciation, it appears that in some hybridizations lock-and-key mechanisms do in fact contribute to RI (Table 1). However, despite the widespread diversity of genital morphologies among many animal taxa, it is clear that genitalia usually do not cause structural lock-and-key RI in the strict sense [4, 9]. In most species crosses where structural lock-and-key has been tested, it seems reasonable to suspect that the criteria for structural isolation were not satisfied—correlated differences of genital morphology between males and females within a species appear to occur much less frequently than the case of substantial diversity of male genital morphology among closely related species, but relatively little diversity of morphology among females of those species. This common sexual asymmetry in the degree of genital morphological divergence suggests the possibility that sensory lock-and-key RI mechanisms, however, could be quite common in limiting gene flow between species [4]. 

Thus, two major challenges face the study of genital evolution and its role in speciation for the near future. The first is to identify the frequency with which RI via genital sensory lock-and-key occurs among different taxa. This might be most easily tested in insect and arachnid species, although the potential for genital sensory lock-and-key RI in some vertebrate systems is currently being explored (B. Langerhans, personal communication, I. Schlupp, personal communication). The second challenge will be to dissect the mechanistic basis—both phenotypically and molecularly—of morphologically induced behaviors or physiological responses that result from genital incompatibilities. This problem will require the availability of sophisticated measurement and molecular tools to manipulate genital morphology, but some experimental systems such as *Carabus *and *Drosophila* appear poised to begin work on determining the sensory consequences of genital morphology and its effect on RI.

## Figures and Tables

**Table 1:** Species hybridizations that display lock-and-key reproductive isolation.

Order	Species hybridized	Lock-and-key mechanism	Reference(s)
Lepidoptera	*Malacosoma franconica* and *M. neustria *	Structural	Standfuss [15]
	*Deilephia porcellus *and *D. elpenor *	Structural	Federley [16]
	*Erebia nivalis* and *E. cassioides *	Sensory	Lorkovic [27]

Araneae	*Misumenops rothi* and *M. gabrielensis *	Structural	Schick [17]
	*Misumenops lepidus* and *M. californicus *	Structural	Schick [17]

Coleoptera	*Carabus iwawakianus* and *C. maiyasanus *	Structural	Sota and Kubota [18]
	*Macrodactylus costulatus*, *M. sylphis*, and* M. sericinus *	Sensory	Eberhard [3]

Polydesmida	*Parafontaria tonominea *sp. A and sp. B	Structural	Tanabe and Sota [21]

Zygoptera	*Sympecma*, *Lestes* (7 species total)	Sensory/structural	Loibl [31]
	*Ischnura elegans* with *Enallagma*, *Platycnemis*,* Sympecma*, *Lestes* (various species)	Sensory/structural	Krieger and Krieger-Loibl [32]
	*Argia*, *Enallagma*, *Ischnura*, *Telebasis *(10 species total)	Sensory/structural	Paulson [33]

Diptera	*Drosophila santomea* and *D. yakuba *	Structural	Kamimura and Mitsumoto [24]
	*Drosophila mauritiana*, *D. sechellia, *and *D. simulans *	Sensory	Robertson [42]
			Cobb et al. [41]
			Coyne [37]
			Price et al. [40]
			Jagadeeshan and Singh [38]
